# Supplementary material for: Evaluating the perceived outcome and impact of an integrated knowledge translation approach in the development of an equity reporting guideline: A cross-sectional survey
Source: PLOS Glob Public Health. 2026 Jan 27;6(1):e0005854. doi: 10.1371/journal.pgph.0005854 (PMC12843522; doi:10.1371/journal.pgph.0005854)
Supplement: S1 Text — S1 Table A. Checklist for reporting of survey studies (CROSS). S1 Text A. Survey Questions. S1 Table B. GRIPP2 reporting checklist. (DOCX) [file pgph.0005854.s001.docx]

**Supplementary material**

**S1 Table A. Checklist for reporting of survey studies (CROSS)**

| **Section/topic** | **Item** | **Item description** | **Reported on page #** |
| --- | --- | --- | --- |
| **Title and abstract** | | |  |
| Title and abstract | 1a | State the word “survey” along with a commonly used term in title or abstract to introduce the study’s design. | 1 |
|  | 1b | Provide an informative summary in the abstract, covering background, objectives, methods, findings/results, interpretation/discussion, and conclusions. | 4 |
| **Introduction** | | |  |
| Background | 2 | Provide a background about the rationale of study, what has been previously done, and why this survey is needed. | 5-6 |
| Purpose/aim | 3 | Identify specific purposes, aims, goals, or objectives of the study. | 6 |
| **Methods** | | |  |
| Study design | 4 | Specify the study design in the methods section with a commonly used term (e.g., cross-sectional or longitudinal). | 6 |
|  | 5a | Describe the questionnaire (e.g., number of sections, number of questions, number and names of instruments used). | 6 |
| Data collection methods | 5b | Describe all questionnaire instruments that were used in the survey to measure particular concepts. Report target population, reported validity and reliability information, scoring/classification procedure, and reference links (if any). | 6 |
|  | 5c | Provide information on pretesting of the questionnaire, if performed (in the article or in an online supplement). Report the method of pretesting, number of times questionnaire was pre-tested, number and demographics of participants used for pretesting, and the level of similarity of demographics between pre-testing participants and sample population. | 7 |
|  | 5d | Questionnaire if possible, should be fully provided (in the article, or as appendices or as an online supplement). | Appendix II |
| Sample characteristics | 6a | Describe the study population (i.e., background, locations, eligibility criteria for participant inclusion in survey, exclusion criteria). | 7-8 |
|  | 6b | Describe the sampling techniques used (e.g., single stage or multistage sampling, simple random sampling, stratified sampling, cluster sampling, convenience sampling). Specify the locations of sample participants whenever clustered sampling was applied. | Not applicable |
|  | 6c | Provide information on sample size, along with details of sample size calculation. | Not applicable |
|  | 6d | Describe how representative the sample is of the study population (or target population if possible), particularly for population-based surveys. | 7-8 |
| Survey  administration | 7a | Provide information on modes of questionnaire administration, including the type and number of contacts, the location where the survey was conducted (e.g., outpatient room or by use of online tools, such as SurveyMonkey). | 7-8 |
|  | 7b | Provide information of survey’s time frame, such as periods of recruitment, exposure, and follow-up days. | 8 |
|  | 7c | Provide information on the entry process:  –>For non-web-based surveys, provide approaches to minimize human error in data entry.  –>For web-based surveys, provide approaches to prevent “multiple participation” of participants. | Not applicable |
| Study preparation | 8 | Describe any preparation process before conducting the survey (e.g., interviewers’ training process, advertising the survey). | 9-10 |
| Ethical considerations | 9a | Provide information on ethical approval for the survey if obtained, including informed consent, institutional review board [IRB] approval, Helsinki declaration, and good clinical practice [GCP] declaration (as appropriate). | 20 |
|  | 9b | Provide information about survey anonymity and confidentiality and describe what mechanisms were used to protect unauthorized access. | 20 |
| Statistical  analysis | 10a | Describe statistical methods and analytical approach. Report the statistical software that was used for data analysis. | 10 |
|  | 10b | Report any modification of variables used in the analysis, along with reference (if available). | Not applicable |
|  | 10c | Report details about how missing data was handled. Include rate of missing items, missing data mechanism (i.e., missing completely at random [MCAR], missing at random [MAR] or missing not at random [MNAR]) and methods used to deal with missing data (e.g., multiple imputation). | 10 |
|  | 10d | State how non-response error was addressed. | Not applicable |
|  | 10e | For longitudinal surveys, state how loss to follow-up was addressed. | Not applicable |
|  | 10f | Indicate whether any methods such as weighting of items or propensity scores have been used to adjust for non-representativeness of the sample. | Not applicable |
|  | 10g | Describe any sensitivity analysis conducted. | Not applicable |
| **Results** | | |  |
| Respondent characteristics | 11a | Report numbers of individuals at each stage of the study. Consider using a flow diagram, if possible. | Not applicable |
|  | 11b | Provide reasons for non-participation at each stage, if possible. | Not applicable |
|  | 11c | Report response rate, present the definition of response rate or the formula used to calculate response rate. | 10 |
|  | 11d | Provide information to define how unique visitors are determined. Report number of unique visitors along with relevant proportions (e.g., view proportion, participation proportion, completion proportion). | Not applicable |
| Descriptive  results | 12 | Provide characteristics of study participants, as well as information on potential confounders and assessed outcomes. | 10-11 |
| Main findings | 13a | Give unadjusted estimates and, if applicable, confounder-adjusted estimates along with 95% confidence intervals and p-values. | Not applicable |
|  | 13b | For multivariable analysis, provide information on the model building process, model fit statistics, and model assumptions (as appropriate). | Not applicable |
|  | 13c | Provide details about any sensitivity analysis performed. If there are considerable amount of missing data, report sensitivity analyses comparing the results of complete cases with that of the imputed dataset (if possible). | Not applicable |
| **Discussion** | | |  |
| Limitations | 14 | Discuss the limitations of the study, considering sources of potential biases and imprecisions, such as non-representativeness of sample, study design, important uncontrolled confounders. | 19 |
| Interpretations | 15 | Give a cautious overall interpretation of results, based on potential biases and imprecisions and suggest areas for future research. | 18 |
| Generalizability | 16 | Discuss the external validity of the results. | 18 |
| **Other sections** | | |  |
| Role of funding source | 17 | State whether any funding organization has had any roles in the survey’s design, implementation, and analysis. | 20 |
| Conflict of interest | 18 | Declare any potential conflict of interest. | 20 |
| Acknowledgements | 19 | Provide names of organizations/persons that are acknowledged along with their contribution to the research. | 20 |

**S1 Text A. Survey Questions.**

1. What proportion of STROBE equity project meetings have you attended? (Kick-off, update, and/or consensus meetings)

Responses?

0-25%

25-50%

50-75%

75%+

1. Which stream(s) were you engaged with? (Please select both if applicable)

Global

Indigenous

Both

1. Overall, how would you describe the extent to which you were engaged in the STROBE equity project?

1 (not at all engaged)

2 (somewhat engaged)

3 (engaged)

4 (very engaged)

5 (totally engaged)

1. How satisfied are you with the level of your engagement with the STROBE equity project?

1 (not at all satisfied)

2 (somewhat satisfied)

3 (satisfied)

4 (very satisfied)

5 (totally satisfied)

1. Which group do you identify with?

Patients, patient caregivers, patient advocates/organizations (Those with lived experience with the condition of interest or who care for or advocate on behalf of those with lived experience)

Payers of health research (Individuals and organizations that fund research projects, such as government funders, industry funders, foundations)

Payers / Purchasers of Health Services (Individuals, organizations and entities that pay for health services)

Peer Reviewed Journal Editors (Those who set journal policy on guidelines and manage the peer review process and editing)

Policymakers (Individuals, organizations and entities that craft public or private policy (on health) at any level of government)

Principal Investigators and all members of the of research team (Individuals, organizations, and associations that conduct or advocate for health research (primary studies and systematic reviews)

Product makers (Individuals working for companies that manufacture pharmaceuticals, medical devices, medical procedures, health technologies, or for profit educational and behavioural packages)

Program managers (Managers/directors who plan, lead, oversee, or deliver any program that provides public health, community services, or clinical care (e.g. budgeting, hiring, staffing, organizing, coordinating, reporting)

Providers (Persons and their professional associations who provide health care in a professional capacity and allowed by regulatory bodies to provide a health care service)

Public (Individuals in the general population of a defined geographic area excluding patients, caregivers, and health professionals living or working with the condition of interest)

1. What is your disciplinary background?

Statistics

Clinical Epidemiology

Sociology

Law

Clinician

Other (please specify)

1. What do you perceive as the benefits, challenges, and impact of explicitly seeking diversity in our team to develop a reporting guideline extension of STROBE equity?

open 1_________

1. Were there any challenges because of the participatory approach (i.e., integrated knowledge translation) used to develop STROBE equity?

open 2_________

1. What do you consider to be the benefits and/or impacts of using a participatory approach (integrated knowledge translation) for STROBE equity?

open3__________

1. Would you have changed anything about the participatory process?

open 4____________

1. What were your motivations for and perceived benefits from participating in the STROBE equity project?

open 5_____________

1. Do you have any additional comments?

open6__________

1. In what country do you live?

Drop-down list of all the countries

1. What is your country of birth?

Drop-down list of all the countries

1. What are your ethnic origins or ancestry? Select all geographic areas from which your ancestors first originated.

Western Europe (e.g., Greece, Sweden, United Kingdom)

Eastern Europe (e.g., Hungary, Poland, Russia)

North Africa (e.g., Egypt, Morocco, Sudan)

Sub-Saharan Africa (e.g., Kenya, Nigeria, South Africa)

West Asia/Middle East (e.g., Iran, Israel, Saudi Arabia)

South and Southeast Asia (e.g., China, Japan, Uzbekistan)

Pacific/Oceania (e.g., Australia, Fiji, Papua New Guinea)

North America (Canada, Mexico, United States)

Central America and Caribbean (e.g., Jamaica, Panama)

South America (e.g., Brazil, Chile, Colombia)

I prefer not to disclose

Other (please specify)

1. How would you identify yourself in terms of ethnicity? Select all groups that apply to you.

Asian or Pacific Islander

Black

Hispanic or Latino/a/x

Indigenous (e.g., North American Indian Navajo, South American Indian Quechua, Aboriginal or Torres Strait Islander)

Middle Eastern or North African

White

I prefer not to disclose

Other (please specify)

1. Select your age range.

18-34

35-54

55-64

65-74

75-84

85-94

95 and above

I prefer not to disclose

1. Which term(s) best describe your current gender identity? Select all that apply.

Agender

Bigender/Multigender

Genderfluid

Genderqueer

Man

Nonbinary

Questioning

Transgender

Two-Spirit

Woman

I prefer not to disclose

Other (please specify)

1. Please tell us your occupation.

open7__________

**S1 Table B. GRIPP2 reporting checklist.**

| **Section and topic** | **Item** | **Reported on page no** |
| --- | --- | --- |
| 1: Aim | Report the aim of PPI in the study | 6 |
| 2: Methods | Provide a clear description of the methods used for PPI in the study | 6 |
| 3: Study results | Outcomes—Report the results of PPI in the study, including both positive and negative outcomes | 6 |
| 4: Discussions and conclusion | Outcomes—Report the results of PPI in the study, including both positive and negative outcomes | 6 |
| 5: Reflections/critical perspective | Comment critically on the study, reflecting on the things that went well and those that did not, so others can learn from this experience | 6 |
