# Supplementary material for: Evaluating the perceived outcome and impact of an integrated knowledge translation approach in the development of an equity reporting guideline: A cross-sectional survey
Source: PLOS Glob Public Health. 2026 Jan 27;6(1):e0005854. doi: 10.1371/journal.pgph.0005854 (PMC12843522; doi:10.1371/journal.pgph.0005854)
Supplement: Attachment — Submitted filename: Peer review comments.docx [file pgph.0005854.s002.docx]

No changes were done to the manuscript as it has not been reviewed yet.

We are resubmitting a previously refused manuscript as is, according to Dr Katherine Kokkinias’s suggestions. The rejection decision was based on the editorial assessment that our submission did not meet the journal’s ethical requirements for human subjects research.

After further communication with the editorial office, we learned that the ethics approval documentation was considered incomplete.

While we had submitted the approval documents from the Bruyère Research Institute, we had forgot to attach documentation from the University of Ottawa Research Ethics Board, despite having obtained full approval from both institutions. The rejection therefore arose from a communication error rather than a lack of ethics approval.

We have now gathered and are prepared to provide the complete set of documents, including the University of Ottawa approval, to demonstrate that all ethical requirements were satisfied prior to conducting the study.

We are grateful to the editors for allowing us the opportunity to clarify and to provide the missing documentation in support of our appeal.
