# Supplementary material for: Evaluating the perceived outcome and impact of an integrated knowledge translation approach in the development of an equity reporting guideline: A cross-sectional survey
Source: PLOS Glob Public Health. 2026 Jan 27;6(1):e0005854. doi: 10.1371/journal.pgph.0005854 (PMC12843522; doi:10.1371/journal.pgph.0005854)
Supplement: Attachment — Submitted filename: Response to reviewers.docx [file pgph.0005854.s003.docx]

Journal Requirements:

1. Please provide additional details regarding participant consent. In the ethics statement in the Methods and online submission information, please ensure that you have specified (1) whether consent was informed and (2) what type you obtained (for instance, written or verbal, and if verbal, how it was documented and witnessed). If your study included minors, state whether you obtained consent from parents or guardians. If the need for consent was waived by the ethics committee, please include this information.

**Response to comment: We have ensured this information is provided in the manuscript**

**Addition to manuscript: “Ethics approval was obtained from the Bruyère Research Institute (M16-21-025) and the University of Ottawa (H-01-22-7671 - OTH-7671) Ethics Boards. All participants provided informed consent before participating in the study by signing the survey form. The study did not include any minors.”**

If you are reporting a retrospective study of medical records or archived samples, please ensure that you have discussed whether all data were fully anonymized before you accessed them and/or whether the IRB or ethics committee waived the requirement for informed consent. If patients provided informed written consent to have data from their medical records used in research, please include this information.

**Response to comment: This does not apply to our study.**

**No changes to the manuscript were made.**

2. Please provide a detailed online Financial Disclosure statement. This is published with the article. It must therefore be completed in full sentences and contain the exact wording you wish to be published.

a) State the initials, alongside each funding source, of each author to receive each grant. For example: “This work was supported by the National Institutes of Health (####### to AM; ###### to CJ) and the National Science Foundation (###### to AM).”
b) State what role the funders took in the study. If the funders had no role in your study, please state: “The funders had no role in study design, data collection and analysis, decision to publish, or preparation of the manuscript.”

For more information, please go to our submission guidelines:
<https://journals.plos.org/globalpublichealth/s/submission-guidelines#loc-financial-disclosure-statement>

**Response to comment: We have ensured that financial disclosures have been detailed in the article.**

**Change to manuscript: “The STROBE-Equity project (VW) was supported by the Canadian Institutes of Health Research (CIHR), grant number 173269. The funder had no role in the study design, the collection, analysis, and interpretation of data or the writing of the article or the decision to submit it for publication. The first author (JB) was funded by the University of Ottawa through the Medical Student Summer Research Program. VW holds an Applied Public Health Research Chair funded by CIHR-PHAC. The funds partially supported this work. The funder had no role in the design, conduct, or reporting of this study.”**

3. Please ensure that the funders and grant numbers match between the Financial Disclosure field and the Funding Information tab in your submission form. Note that the funders must be provided in the same order in both places as well.

**Response to comment: We have ensured that the funders and grant numbers match between the Financial Disclosure field and the Funding Information tab in our submission form.**

4. We note that you have indicated that there are restrictions to data sharing for this study. For studies involving human research participant data or other sensitive data, we encourage authors to share de-identified or anonymized data. However, when data cannot be publicly shared for ethical reasons, we allow authors to make their data sets available upon request. For information on unacceptable data access restrictions, please see <http://journals.plos.org/globalpublichealth/s/data-availability#loc-unacceptable-data-access-restrictions>.

Before we proceed with your manuscript, please address the following prompts:

a) If there are ethical or legal restrictions on sharing a de-identified data set, please explain them in detail (e.g., data contain potentially identifying or sensitive patient information, data are owned by a third-party organization, etc.) and who has imposed them (e.g., a Research Ethics Committee or Institutional Review Board, etc.). Please also provide contact information for a data access committee, ethics committee, or other institutional body to which data requests may be sent.

b) If there are no restrictions, please upload the minimal anonymized data set necessary to replicate your study findings to a stable, public repository and provide us with the relevant URLs, DOIs, or accession numbers. Please see <http://www.bmj.com/content/340/bmj.c181.long> for guidelines on how to de-identify and prepare clinical data for publication. For a list of recommended repositories, please see <https://journals.plos.org/globalpublichealth/s/recommended-repositories>. You also have the option of uploading the data as Supporting Information files, but we would recommend depositing data directly to a data repository if possible.

Please update your Data Availability statement in the submission form accordingly.

**Response to comment: We are unable to share the data with the public given the low number of participants (n = 19) and collection of information that would make the participants identifiable. Our data availability has not changed.**

5. Please provide separate figure files in .tif or .eps format only and remove any figures embedded in your manuscript file. Please also ensure that all files are under our size limit of 10MB. Please leave the figure captions in the manuscript.

For more information about how to convert your figure files please see our guidelines: <https://journals.plos.org/globalpublichealth/s/figures>

**Response to comment: We have submitted the figures separately.**

6. We notice that your supplementary materials are included in the manuscript file. Please remove them and upload them with the file type 'Supporting Information'. Please ensure that each Supporting Information file has a legend listed in the manuscript before or after the references list.

**Response to comment: We have submitted the supplementary material separately.**

7. Some material included in your submission may be copyrighted. According to PLOS’s copyright policy, authors who use figures or other material (e.g., graphics, clipart, maps) from another author or copyright holder must demonstrate or obtain permission to publish this material under the Creative Commons Attribution 4.0 International (CC BY 4.0) License used by PLOS journals. Please closely review the details of PLOS’s copyright requirements here: PLOS Licenses and Copyright. If you need to request permissions from a copyright holder, you may use PLOS's Copyright Content Permission form.

Please respond directly to this email or email the journal office and provide any known details concerning your material's license terms and permissions required for reuse, even if you have not yet obtained copyright permissions or are unsure of your material's copyright compatibility.

Potential Copyright Issues:

Figure 2: please (a) provide a direct link to the base layer of the map (i.e., the country or region border shape) and ensure this is also included in the figure legend; and (b) provide a link to the terms of use / license information for the base layer image or shapefile. We cannot publish proprietary or copyrighted maps (e.g. Google Maps, Mapquest) and the terms of use for your map base layer must be compatible with our CC-BY 4.0 license.

Note: if you created the map in a software program like R or ArcGIS, please locate and indicate the source of the basemap shapefile onto which data has been plotted.

If your map was obtained from a copyrighted source please amend the figure so that the base map used is from an openly available source. Alternatively, please provide explicit written permission from the copyright holder granting you the right to publish the material under our CC-BY 4.0 license.

Please note that the following CC BY licenses are compatible with PLOS license: CC BY 4.0, CC BY 2.0 and CC BY 3.0, meanwhile such licenses as CC BY-ND 3.0 and others are not compatible due to additional restrictions.

If you are unsure whether you can use a map or not, please do reach out and we will be able to help you. The following websites are good examples of where you can source open access or public domain maps: 
* U.S. Geological Survey (USGS) - All maps are in the public domain. (<http://www.usgs.gov>) 
* PlaniGlobe - All maps are published under a Creative Commons license so please cite “PlaniGlobe, <http://www.planiglobe.com>, CC BY 2.0” in the image credit after the caption. (<http://www.planiglobe.com/?lang=enl>) 
* Natural Earth - All maps are public domain. (<http://www.naturalearthdata.com/about/terms-of-use/>)

**Response to comment: We generated the map using Microsoft excel. Excel’s built-in map tool uses a proprietary basemap without a citable open license. To comply with CC-BY requirements, we have removed the map.**

8. If the reviewer comments include a recommendation to cite specific previously published works, please review and evaluate these publications to determine whether they are relevant and should be cited. There is no requirement to cite these works unless the editor has indicated otherwise.

**Response to comment: Thank you.**

[Note: HTML markup is below. Please do not edit.]

Reviewers' comments:

Reviewer's Responses to Questions

**Comments to the Author**

1. Does this manuscript meet PLOS Global Public Health’s [publication criteria](https://track.editorialmanager.com/CL0/https:%2F%2Fjournals.plos.org%2Fglobalpublichealth%2Fs%2Fcriteria-for-publication/1/010f019a52b3e314-b5f44065-8414-4864-a8a2-1a899b983ab3-000000/-TuCoH_zoaYSfVakOoYJg1RNnq8owtFvDJprcz50RRM=236)? Is the manuscript technically sound, and do the data support the conclusions? The manuscript must describe methodologically and ethically rigorous research with conclusions that are appropriately drawn based on the data presented.

Reviewer #1: Yes

Reviewer #2: Partly

2. Has the statistical analysis been performed appropriately and rigorously?

Reviewer #1: N/A

Reviewer #2: Yes

3. Have the authors made all data underlying the findings in their manuscript fully available (please refer to the Data Availability Statement at the start of the manuscript PDF file)?

The [PLOS Data policy](https://track.editorialmanager.com/CL0/https:%2F%2Fjournals.plos.org%2Fglobalpublichealth%2Fs%2Fdata-availability/1/010f019a52b3e314-b5f44065-8414-4864-a8a2-1a899b983ab3-000000/IzqBchNFpaKAZvleu5uFJEVGgK61y1Elz86R0Kxwrus=236) requires authors to make all data underlying the findings described in their manuscript fully available without restriction, with rare exception. The data should be provided as part of the manuscript or its supporting information, or deposited to a public repository. For example, in addition to summary statistics, the data points behind means, medians and variance measures should be available. If there are restrictions on publicly sharing data—e.g. participant privacy or use of data from a third party—those must be specified.

Reviewer #1: No

Reviewer #2: Yes

4. Is the manuscript presented in an intelligible fashion and written in standard English?

PLOS Global Public Health does not copyedit accepted manuscripts, so the language in submitted articles must be clear, correct, and unambiguous. Any typographical or grammatical errors should be corrected at revision, so please note any specific errors here.

Reviewer #1: Yes

Reviewer #2: Yes

5. Review Comments to the Author

Please use the space provided to explain your answers to the questions above. You may also include additional comments for the author, including concerns about dual publication, research ethics, or publication ethics. (Please upload your review as an attachment if it exceeds 20,000 characters)

Reviewer #1: The paper is well-written and covers a relevant topic. The minor adjustment I suggest is related to the Strenghts and Limitations section, rows 588-590: "While the online survey limited the in depth probing characteristic of interview studies, it proved an efficient and appropriate method for gathering perceptions from a large, geographically dispersed, and diverse international team, aligning with our aim to broadly evaluate the IKT process (25).". Since only four respondents lived in LMIC's, there is indeed a limitation in stating that the method is appropriate from gathering perceptions from geographical disperse and diverse teams (i.e. 5 out of 9 members from South America and Africa didn't respond and we don't understand exaclty why). That being said, while the survey seems a promising method, you could explore whether translation of the survey or other inclusivity methods would allow for broader Global South participation.

**Response to comment: We agree that the limited number of respondents from LMICs limits our ability to conclude that our method fully captured perspectives from geographically diverse team members. We have revised the Limitations section to explicitly acknowledge that overall participation was low, the low response rate did not appear to be disproportionately driven by any specific geographic region, we were not able to determine reasons for non-response but this could investigated in future studies.**

**Change to manuscript: “Our response rate of 46% is a limitation. However, the representation of academic and knowledge users team members and geographic diversity was reflective of our overall team and we did not detect a disproportionate lack of participation by geographical location. Nonetheless, the small number of LMIC respondents limits the extent to which the survey captured perspectives from LMICs. Future studies could be conducted to investigate reasons for non-response and suggest potential solutions. While the online survey was limited in its ability to provide in-depth information compared to interview studies, it proved an efficient and appropriate method for gathering perceptions from a large, geographically dispersed, and diverse international team, aligning with our aim to broadly evaluate the IKT process (25).”**

Finally, while the authors didn't provide access to full data, they justified by citing confidentiality purposes. Given that the team is not big, access to full data could foster identification of respondents and all data from the survey was summarized so I believe the justification provided is enough.

**Response to comment: thank you.**

Reviewer #2: 1. The paper presents an evaluation of the IKT methods for a particular project based on a survey of members of the IKT team. The survey and descriptive statistics provided are sound and overall, I am not sure they fully support the conclusions. The survey is of the perception of the team members of the impact of the IKT approach, and the process aspects that worked and didn’t work. But the actual use impact of the IKT approach is not assessed. For example, the participants report anticipates use of the guideline extension but there is no discussion if this was observed, however it seems to be reported as if this is occurring. It would be interesting to know if it is indeed occurring. But no doubt there are time constraints of the project, as it will take some time to know if people are using the guideline extension. Perhaps a more accurate representation of this evaluation is that it is a process evaluation. Then the findings and conclusions can focus on the implications of what worked or didn’t work for this type of IKT team. Which is present for sure, just could expand on that more. I also would have liked to see the evaluation be guided by some of the theories or frameworks in the literature, this might have helped with the more specific/accurate framing of the impact. For example, there is a community engagement assessment tool based on the REAP model that might have been useful to integrate into the analysis. (<https://research.ucalgary.ca/toolkit>) Or from the D&I literature, something like the RE-AIM might have helped to shape the assessment and analysis. Or perhaps another theory of change – if the team was using a theory of change they could present that here and what they know so far. The governance model presented early on might be interpreted this way? The findings could be considered in light of that perhaps?

**Response to comment: Thank you for your thoughtful comment. We agree that our study is best described as a process evaluation. We have clarified the aim in abstract, methods and discussion to reflect that.**

**Change to manuscript: Please see changes line 130-132, 150-156, 241-243, 273, 537-544.**

**We have also revised the text to make it clear that the participant’s views about dissemination and uptake reflect anticipated or perceived impact and that the actual use of the guideline is not yet assessed.**

**Change to manuscript: lines 144-149, 480-487**

**To situate the evaluation more explicitly within an established conceptual model, we now reference the Collaborative Research Framework, which guided both the design and conduct of the project. As noted in our protocol, this framework has previously been used to support integrated knowledge translation processes in guideline-focused research and was selected for its pragmatic emphasis on producing evidence that is useful and actionable for knowledge users (e.g., practitioners, policy actors, and journal editors). The reviewer’s suggestion that our earlier governance model (Figure 1) could serve a similar interpretive function aligns closely with our intent; we now make this connection explicit in the revised text.**

**Change to manuscript: lines 344-353**

**“We applied the Collaborative Research Framework to guide our the evaluation of our IKT approach (21). This framework accommodated formal and structured partnering between knowledge users and researchers in the preparation, planning, and conduct stages of the empirical studies informing the STROBE-Equity reporting guideline. The framework assumes that IKT is facilitated when team members are prepared for ongoing, iterative knowledge exchange and shared learning. Consistent with this, our approach involved two core components: 1) establishing guiding features for co-production through an equity-driven governance structure, and 2) defining and operationalizing research activites that enabled joint production of relevant, usable and equitable evidence. Our selection for this framework was based on previous experience with the framework to guide reporting guideline-focused research involving diverse knowledge users and we utilized it as the conceptual foundation for our IKT approach, as cited in our reporting guideline protocol (4, 22).”**

2. Descriptive statistics are appropriate. 3. No concerns

**Response to comment: thank you.**

4. The manuscript is clear and well written. There are a couple of typos here and there but overall, no concerns here. One overall comment is to make it clearer what you are (and not) doing with this paper. In line 218/19 you state the purpose of the paper. But I don’t know that is totally accurate to what you report. Do you really assess how the IKT approach reflected JEDI? My take away was more around (similar to comment 1) if the IKT approach used in the project was effective? What you learned about how to use it more effectively to be JEDI. And given the IKT focus, I wonder if the balance of background should lean a little more to IKT?

**Response to comment: thank you. As mentioned in the previous comment, our evaluation examined team members’ perceptions of the benefits, challenges, and potential impact of the IKT approach used during development of the guideline. This included their perceptions of how efforts to reflect JEDI principles were experienced as this was central to our IKT approach. We have clarified this throughout the manuscript. Please see response to previous comments.**
